# Supplementary material for: Reconstructing the three-dimensional architecture of extrachromosomal DNA with ec3D
Source: Nat Commun. 2025 Dec 20;17:894. doi: 10.1038/s41467-025-67614-7 (PMC12830899; doi:10.1038/s41467-025-67614-7)
Supplement: Supplementary file 1 — Supplementary Information [file 41467_2025_67614_MOESM1_ESM.pdf]

Supplementary Figures for Reconstructing the three-dimensional architecture of extrachromosomal DNA with ec3D

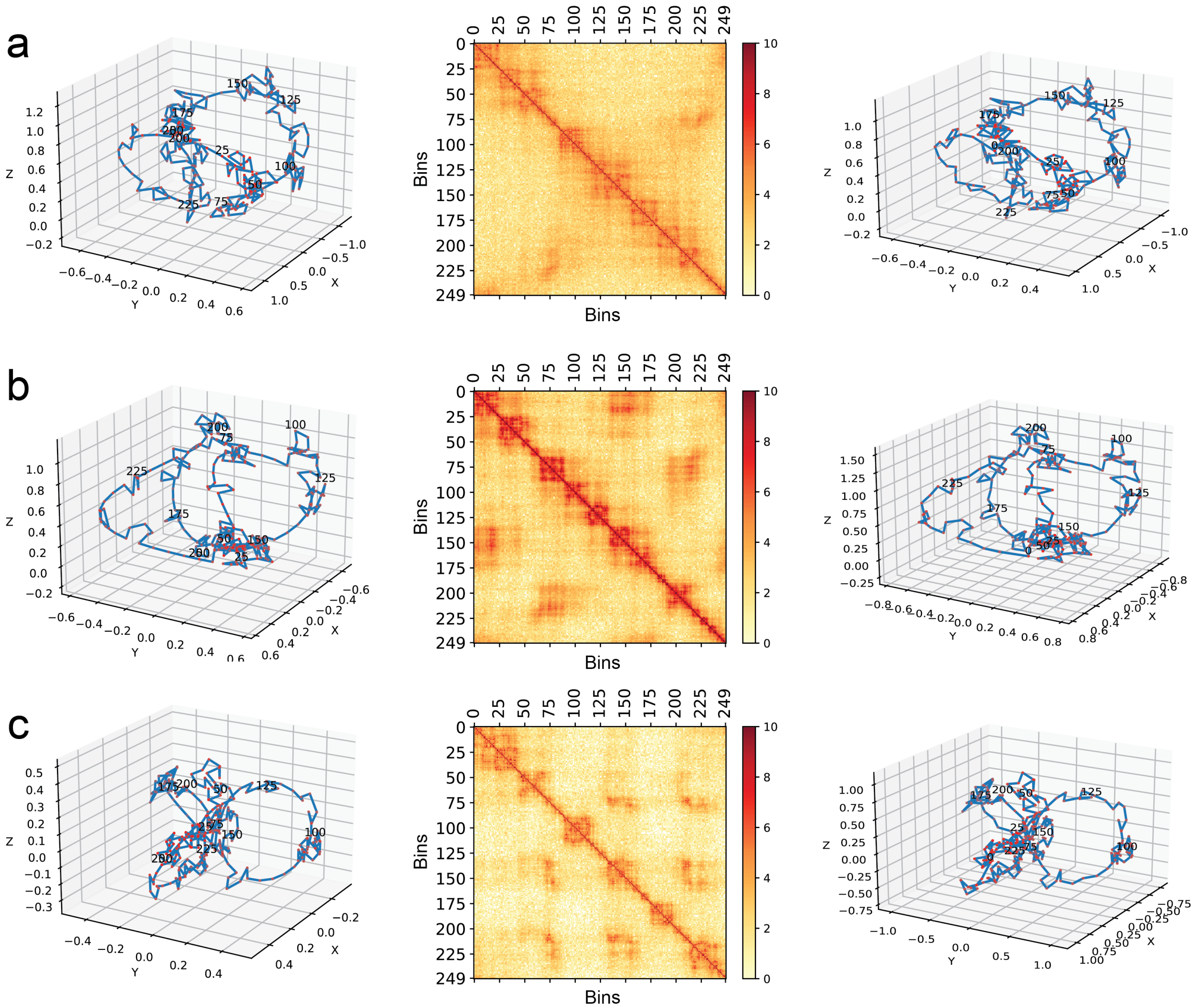

Supplementary Figure 1: Simulating samples with topological constrictions. a, A simulated structure with k=1 topological constriction and its corresponding expanded Hi-C data and reconstructed structure. **b,** k=2. **c,** k=3.

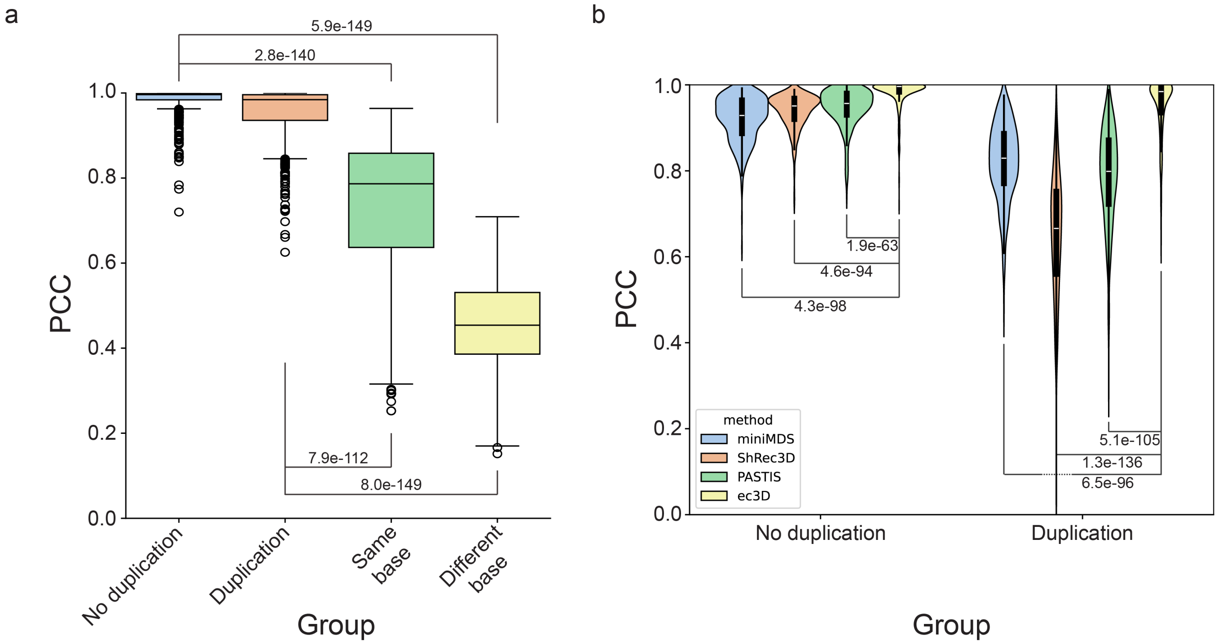

Supplementary Figure 2: PCC-metric simulation test and tool comparison results. a, PCC-metric simulation test results in 4 different groups: **No duplication** - ground-truth versus reconstructed structures without duplication; **Duplication** - ground-truth versus reconstructed structures with duplication; **Same base** - random pairs of structures with the same base structure; **Different base** - random pairs of structures with different base structures. Each group has 450 pairs of samples. P-values were calculated using one-sided Wilcoxon rank-sum test for two samples. Center lines in the box plots indicate the median, boxes represent the interquartile range (IQR) from the 25th to the 75th percentile, whiskers extend to the minimum and maximum values within 1.5 times the IQR. **b,** Violin plots showing the PCC comparisons between ec3D and other methods. Box plots within each violin indicate the median, interquartile range (IQR) from the 25th to the 75th percentile, and whiskers extending to the minimum and maximum values within 1.5 times the IQR. P-values were calculated using one-sided Wilcoxon rank-sum test for two samples. Source data are provided as a Source Data file.

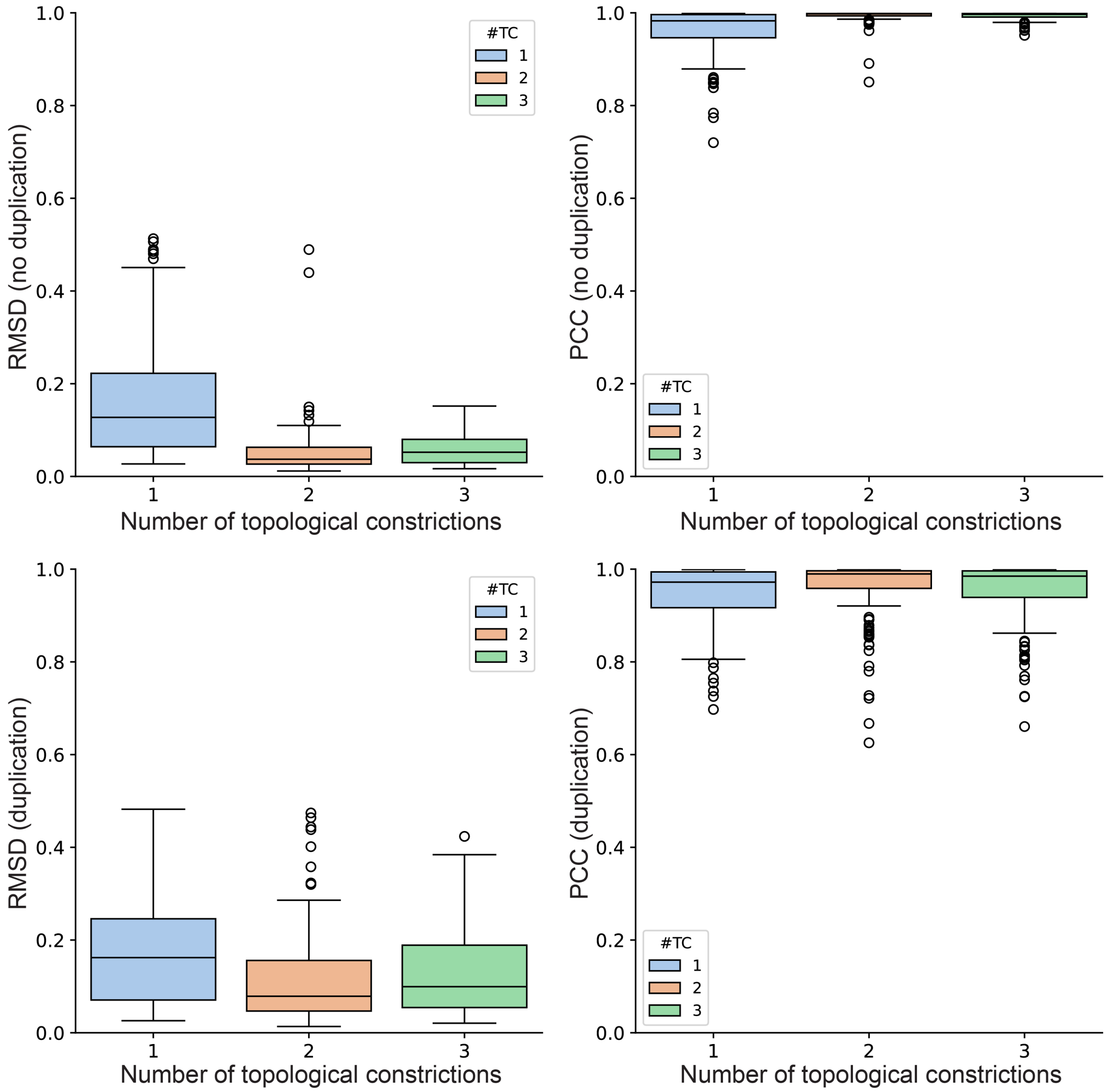

Supplementary Figure 3: Simulation test results grouped by the number of topological constrictions. The RMSD and PCC values were calculated by comparing ground truth and reconstructed structures in simulated data. Each group ($k = 1, 2, 3$ topological constrictions) has 150 simulated samples, with varying local folds and α. Center lines in the box plots indicate the median, boxes represent the interquartile range (IQR) from the 25th to the 75th percentile, whiskers extend to the minimum and maximum values within 1.5 times the IQR. Source data are provided as a Source Data file.

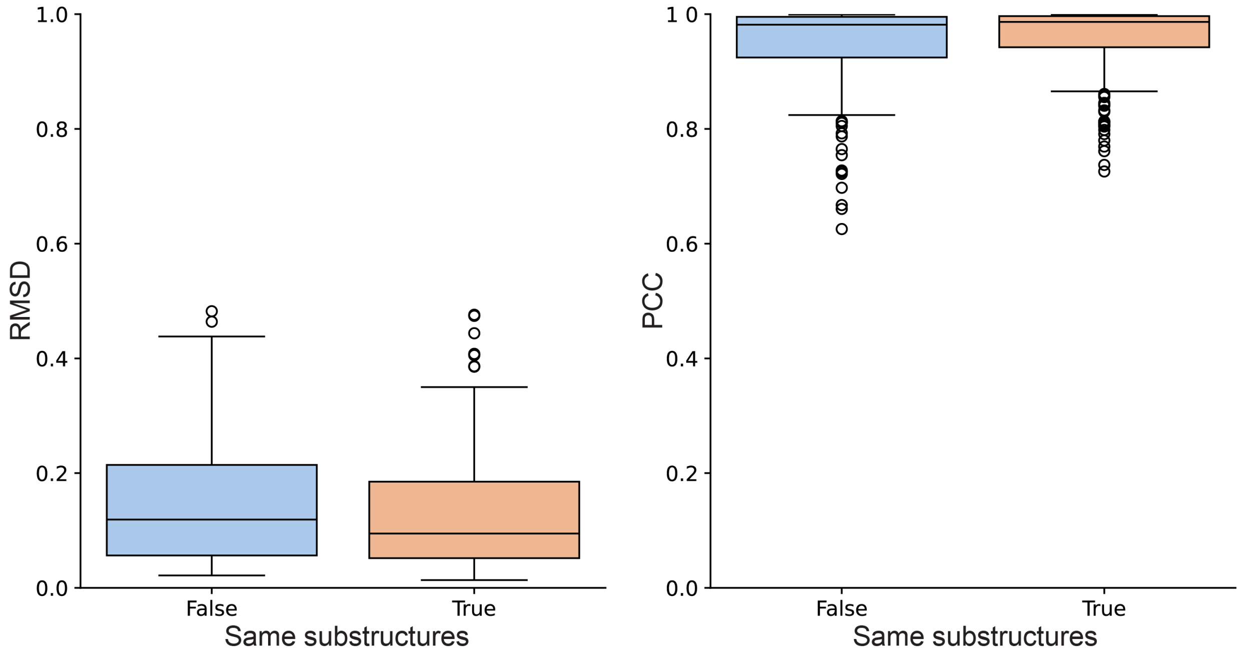

Supplementary Figure 4: Results of simulation tests with duplicated segments that have the same or different local substructures. Each group (same/different local substructure) has 225 simulated samples, with varying number of topological constrictions, local folds and α. Center lines in the box plots indicate the median, boxes represent the interquartile range (IQR) from the 25th to the 75th percentile, whiskers extend to the minimum and maximum values within 1.5 times the IQR. Source data are provided as a Source Data file.

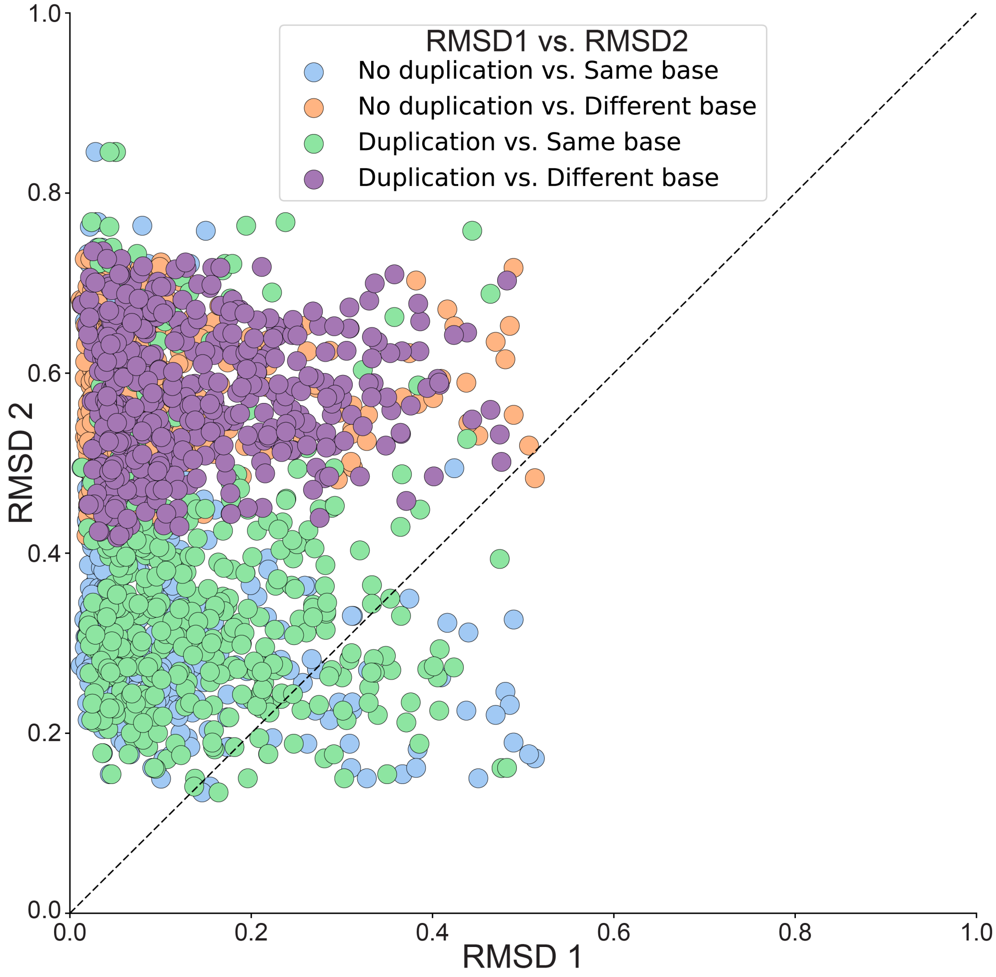

Supplementary Figure 5: RMSD values of ground truth versus reconstructed structures (RMSD1) and RMSD values of ground truth versus random structures (RMSD2) in simulation tests. A data point at the top left of the dashed line indicates that the reconstructed structure is more similar to the ground truth than a random structure. Source data are provided as a Source Data file.

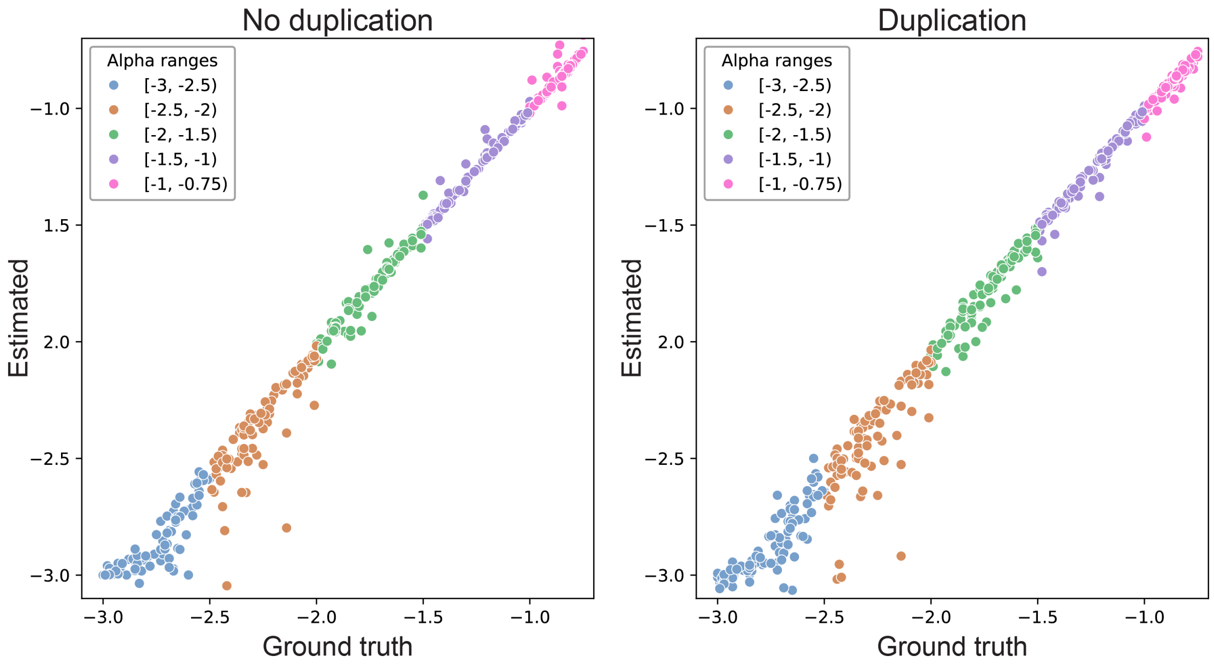

Supplementary Figure 6: Estimated α values versus ground truth α values in simulation tests. Source data are provided as a Source Data file.

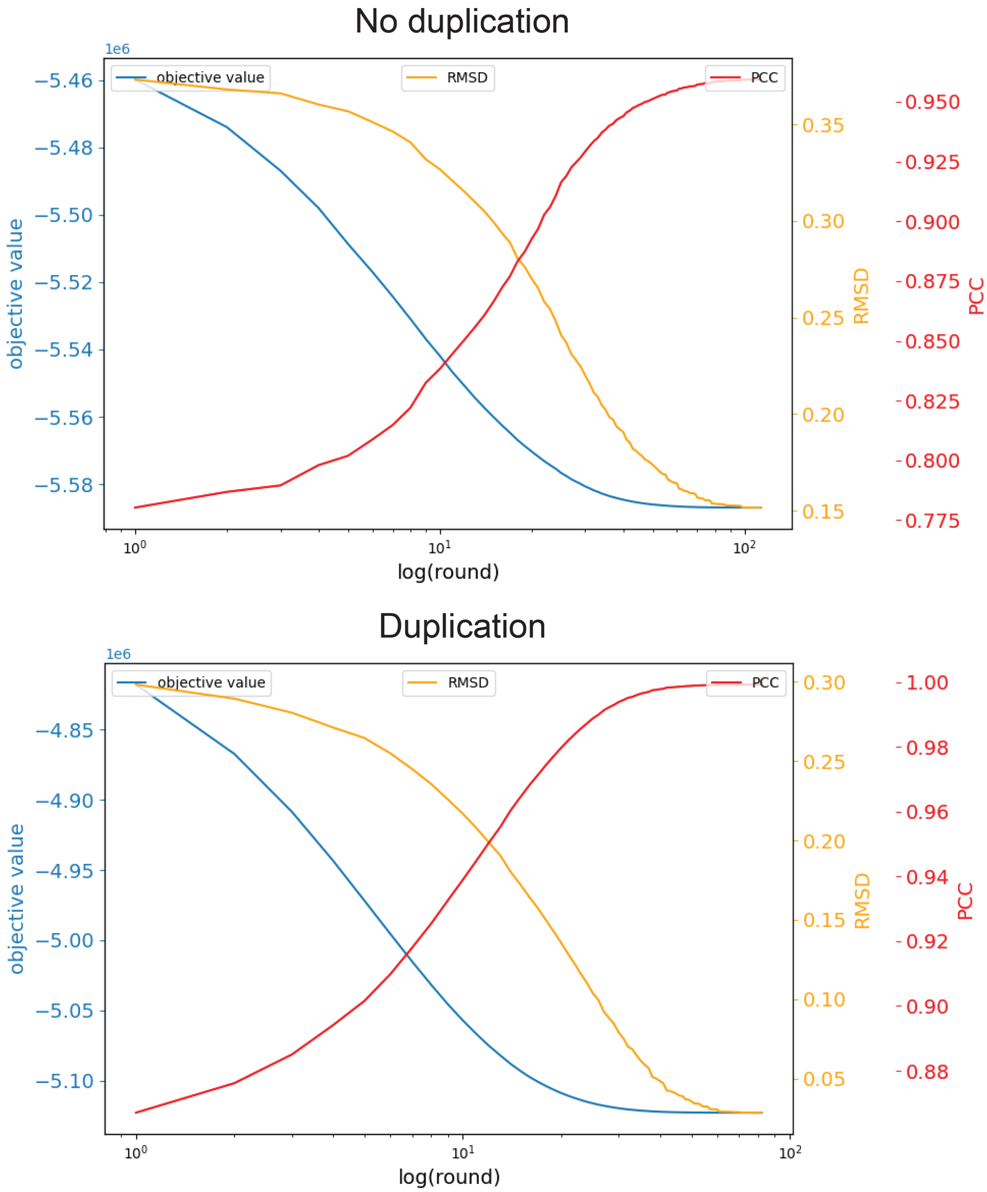

Supplementary Figure 7: Changes of objective values, RMSD, and PPC, during Poisson optimization on two simulated samples (without and with duplication). X-axis indicates the number of iterations (rounds).

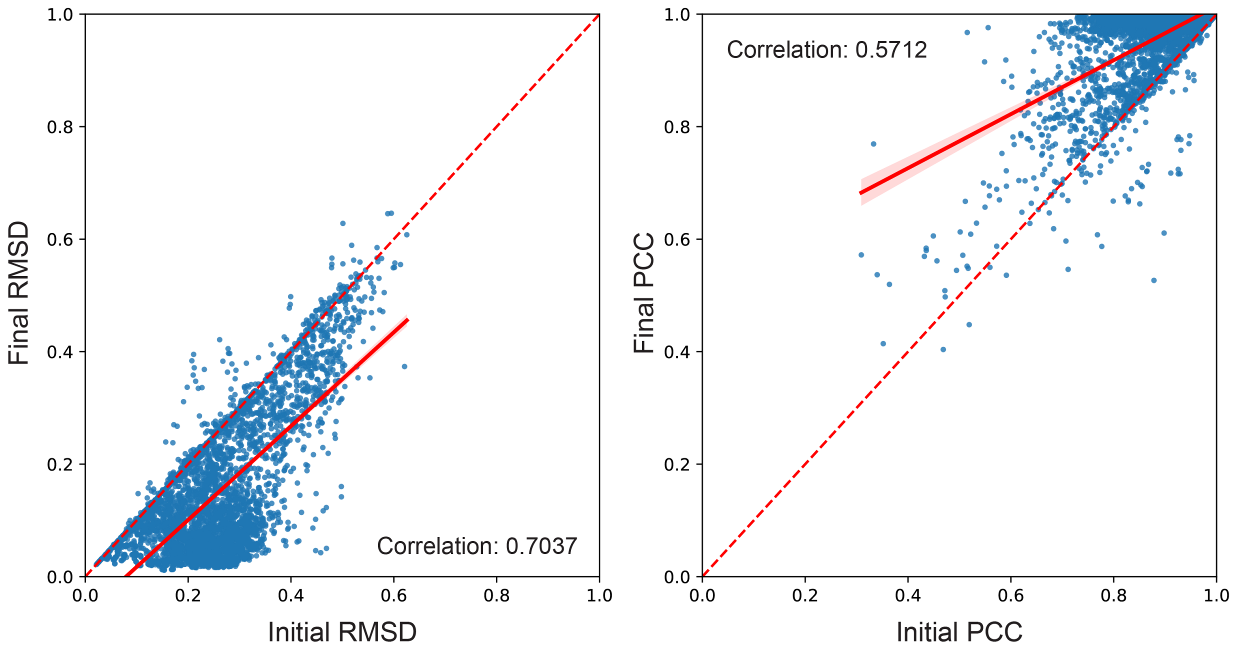

Supplementary Figure 8: Final RMSD (and PCC) values versus initial RMSD (and PCC) values in simulation tests. The majority of RMSD and PCC values were improved through optimization, showing smaller RMSD and larger PCC compared with initial values.

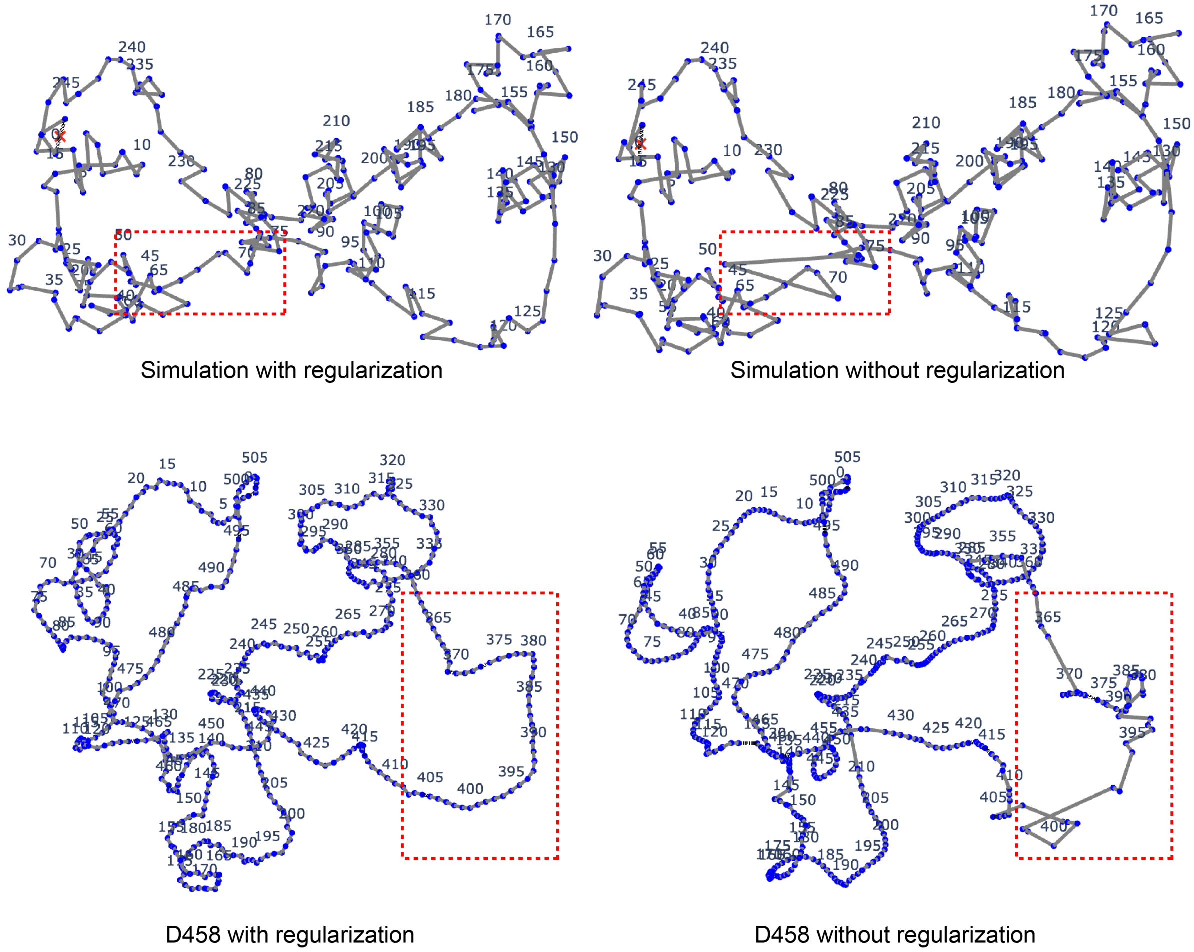

Supplementary Figure 9: 3D structures of a simulated structure (top) and D458 ecDNA (bottom) reconstructed with and without regularization. The red dashed boxes indicate the impact of regularization on the spatial distances of adjacent bins.

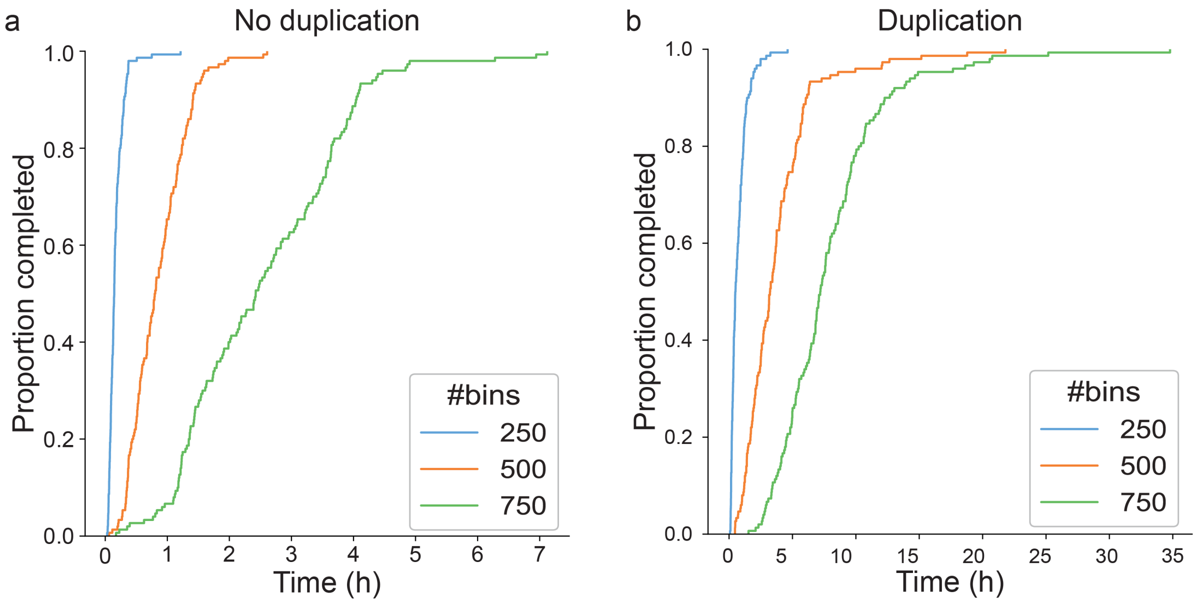

Supplementary Figure 10: Running time for reconstructing structures of different sequence lengths without duplication (a) and with duplication (b). The sequence length is measured by the number of fixed-resolution bins. 150 samples were simulated for each category, resulting in 900 samples in total. The y-axis in the line plots represents the proportion of samples whose reconstruction was completed by a specific time point. Source data are provided as a Source Data file.

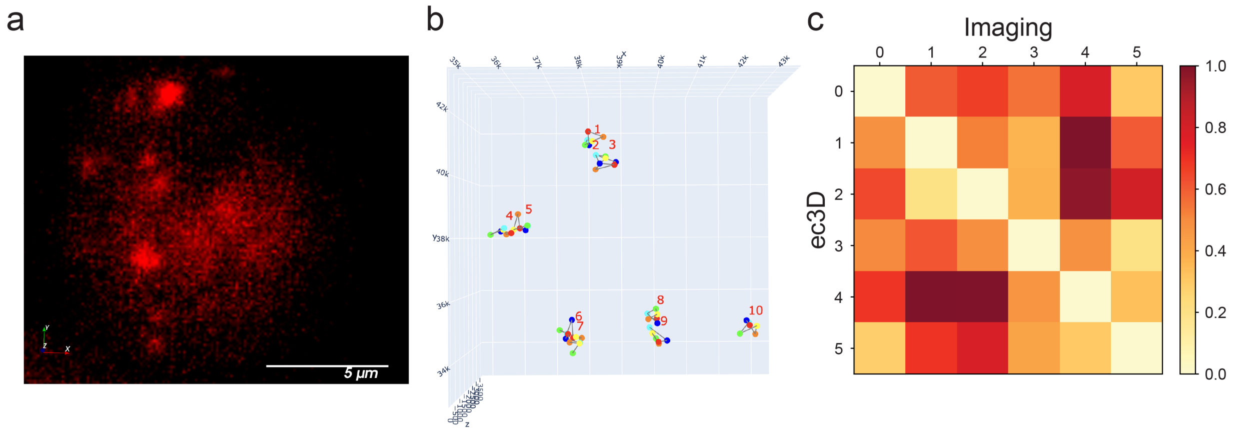

Supplementary Figure 11: Sequential OligoSTORM imaging on *MDM2* ecDNAs in TR14. **a,** Reference image with signals of *MDM2* ecDNA in a single cell. **b,** Verified cliques of probe centroids of 10 ecDNAs in 3D space. **c,** Comparison of the pairwise spatial distances of 200kbp bins obtained by ec3D (lower triangle) and by OligoSTORM imaging (upper triangle) averaged across 10 imaged ecDNAs. Color scale represents normalized spatial distances ranging from 0 (yellow) to 1 (red). Source data are provided as a Source Data file.

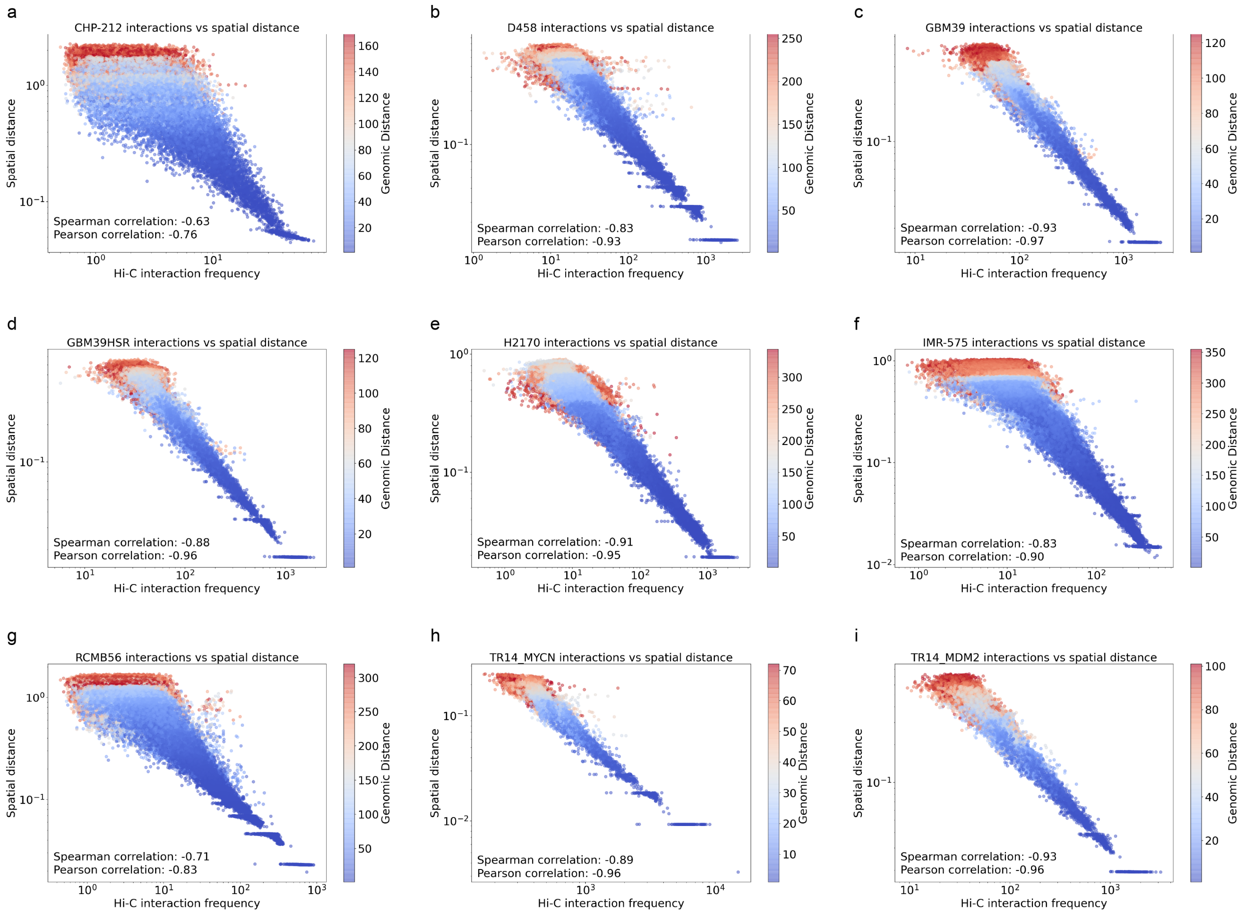

Supplementary Figure 12: Correlation between Hi-C interaction frequencies (x-axis) and spatial (Euclidean) distances (y-axis), from CHP-212 (a), D458 (b), GBM39 (c), GBM39 HSR (d), H2170 (e), IMR-5/75 HSR (f), RCMB56 (g), TR14 *MYCN* (h), and TR14 *MDM2* (i). Color gradient representing the genomic distance (in the number of 5kbp bins) was overlaid on each scatter plot point, with warmer colors indicating shorter genomic distances and cooler colors indicating longer distances.

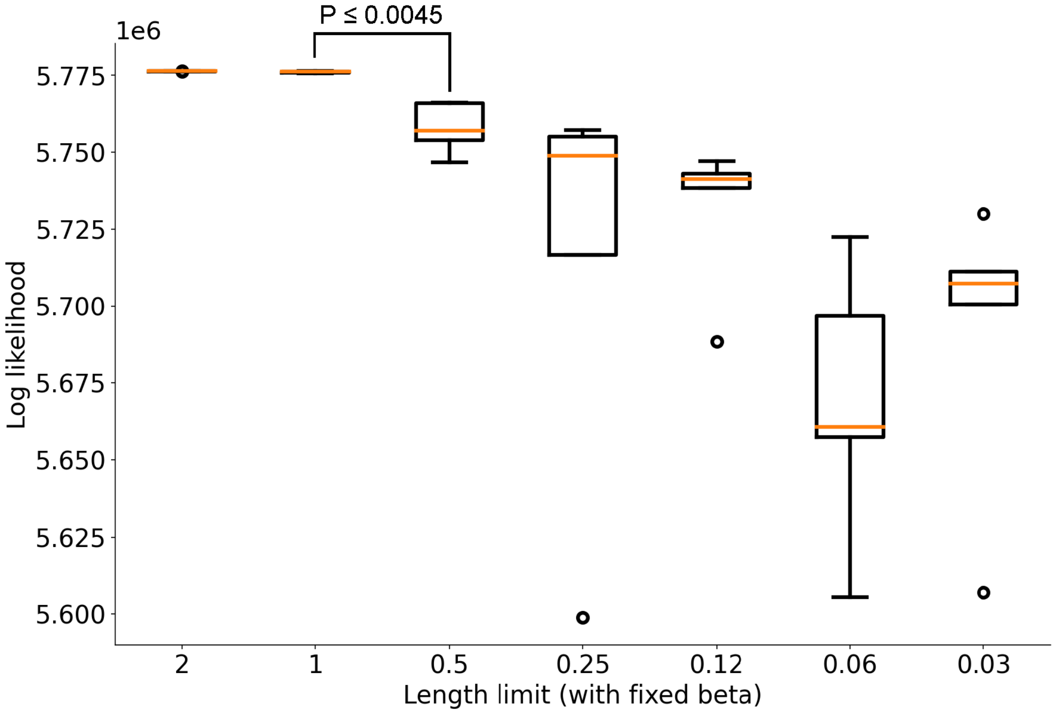

Supplementary Figure 13: Optimal values of ec3D's objective function (y-axis) for RCMB56 when fixing the scaling parameter and limiting the maximum range of the first axis (x-axis). A box plot describing the 5 final objective values with randomly initialized X was made for each length limit ranging from 0.03 to 2. The other two axes remain in the default range [-1, 1]. P-value was calculated using one-sided Wilcoxon rank-sum test for two samples. Center lines (orange) indicate the median, boxes (black) represent the interquartile range (IQR) from the 25th to the 75th percentile, whiskers extend to the minimum and maximum values within 1.5 times the IQR. Source data are provided as a Source Data file.

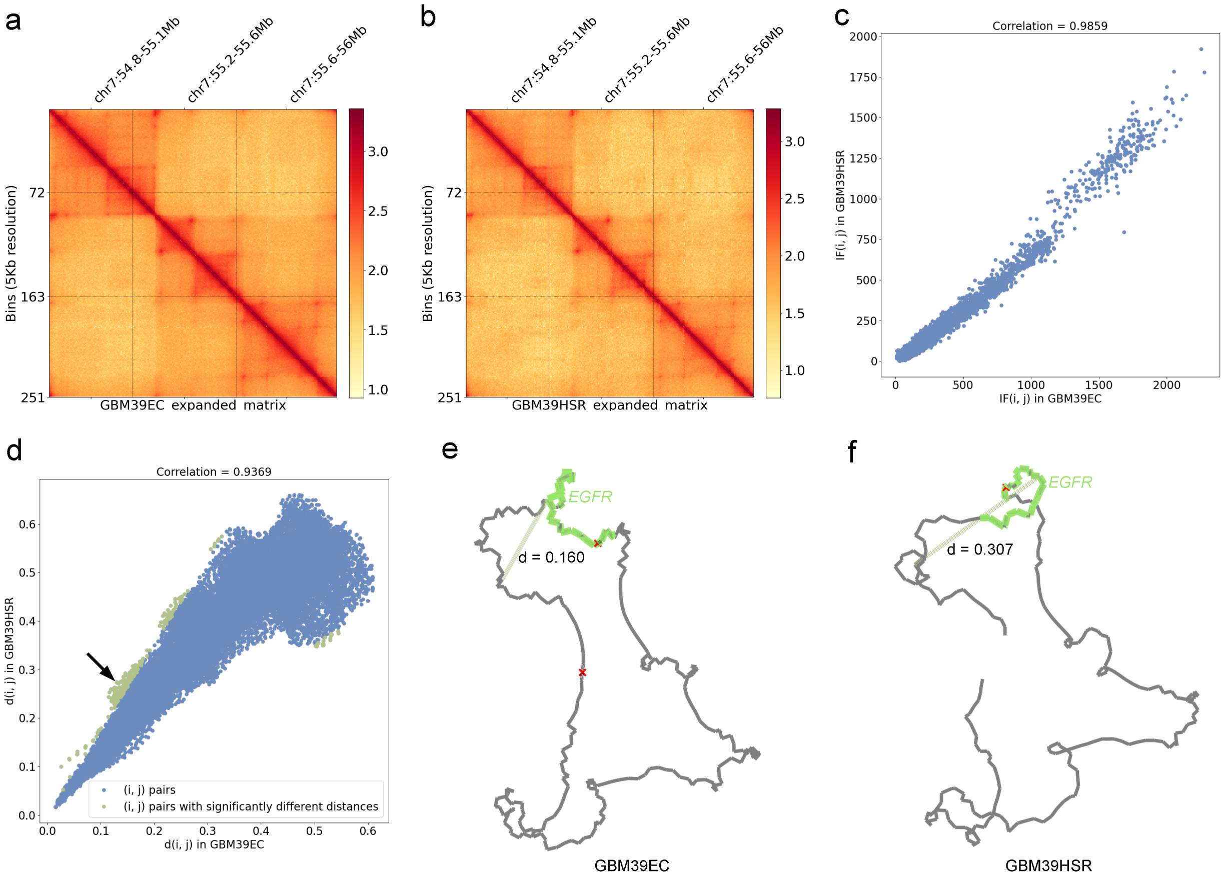

Supplementary Figure 14: Similarities of ecDNA and HSR structures from isogenic cell lines GBM39 and GBM39HSR. a, b, c, normalized Hi-C interaction frequencies and their correlations. x-axis: interaction frequency of GBM39EC; y-axis: the corresponding interaction frequency of GBM39HSR. Color scale represents $\log_{10}$-transformed normalized interaction frequencies. **d,** Euclidean distances between each pair of 5-kb bins in ec3D reconstructions. x-axis: Euclidean distances on GBM39EC structure; y-axis: the corresponding distances on GBM39HSR structure. Bin pairs showed significantly larger or smaller distances between the two structures marked in green. The black arrow points to the example bin pair in panels **e** and **f**. **e, f,** ec3D reconstructions of GBM39 ecDNA and GBM39 HSR, with an example of notable differences indicated by the green lines. Source data are provided as a Source Data file.

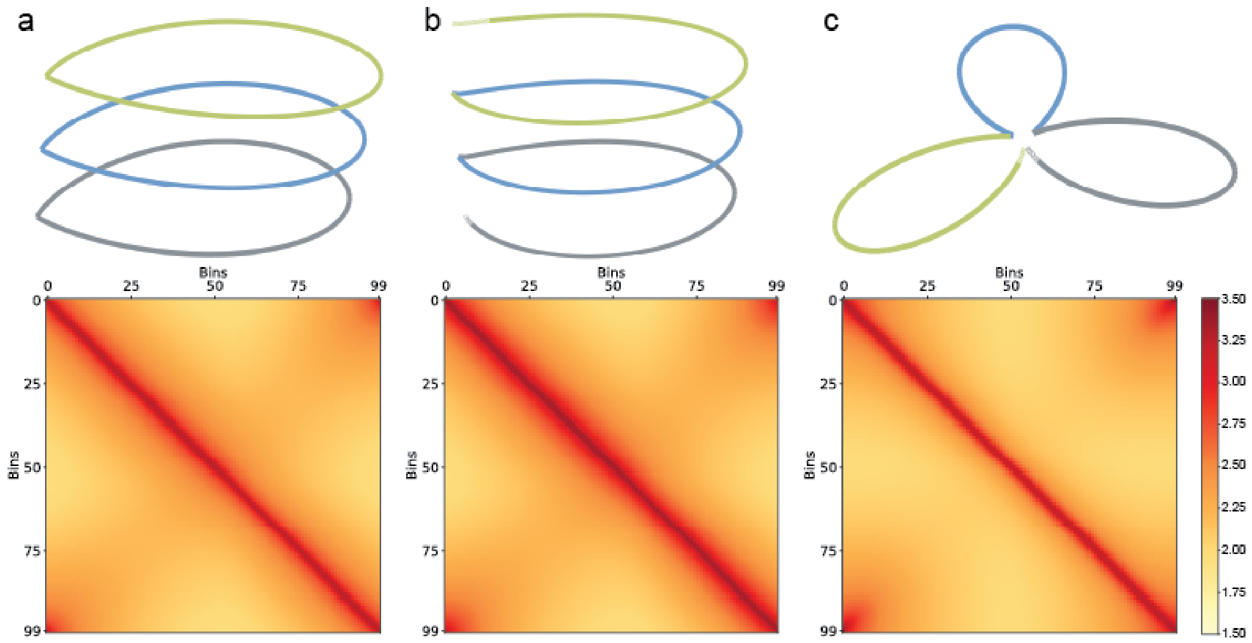

Supplementary Figure 15: EcDNA and potential HSR models. a, A stacked model of ecDNA with the collapsed matrix. **b,** A 'spring-like' HSR model with the collapsed matrix has signals very similar to the stacked ecDNA. **c,** A 'petal-like' HSR model with the collapsed matrix also contains end-to-end interactions but has fewer off-diagonal signals. Each color in the structures represents one copy of duplication.

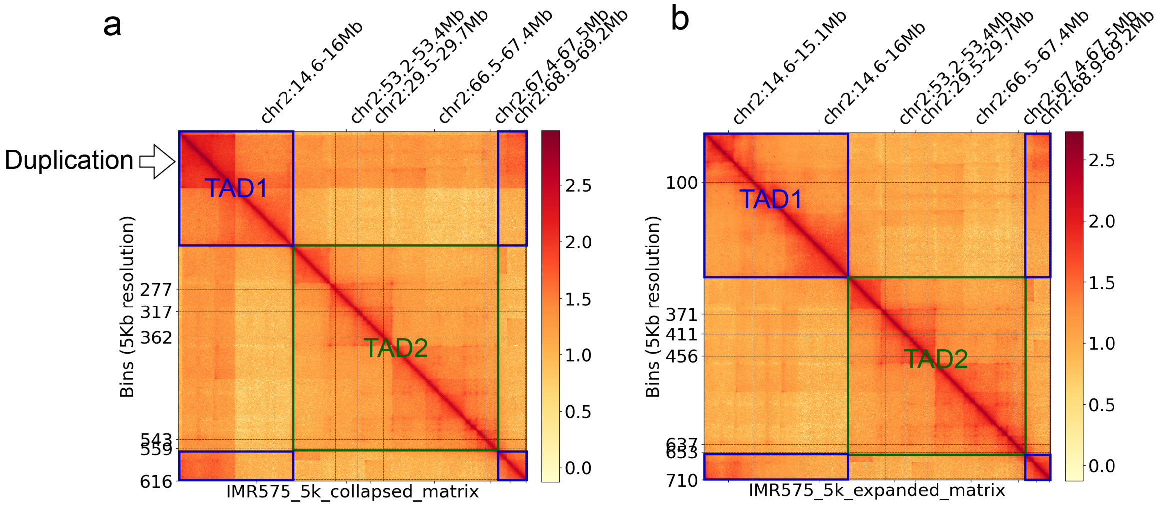

Supplementary Figure 16: Collapsed matrix (a) and expanded matrix (b) of IMR-5/75 HSR. Both matrices display a two-TAD structure, where TAD1 incorporates a joining of the last and the first segment of the cyclic structure, supporting a tandem duplication model of HSR. Color scale represents $\log_{10}$-transformed normalized interaction frequencies. Ec3D reconstruction and the expanded matrix clarify the first TAD.

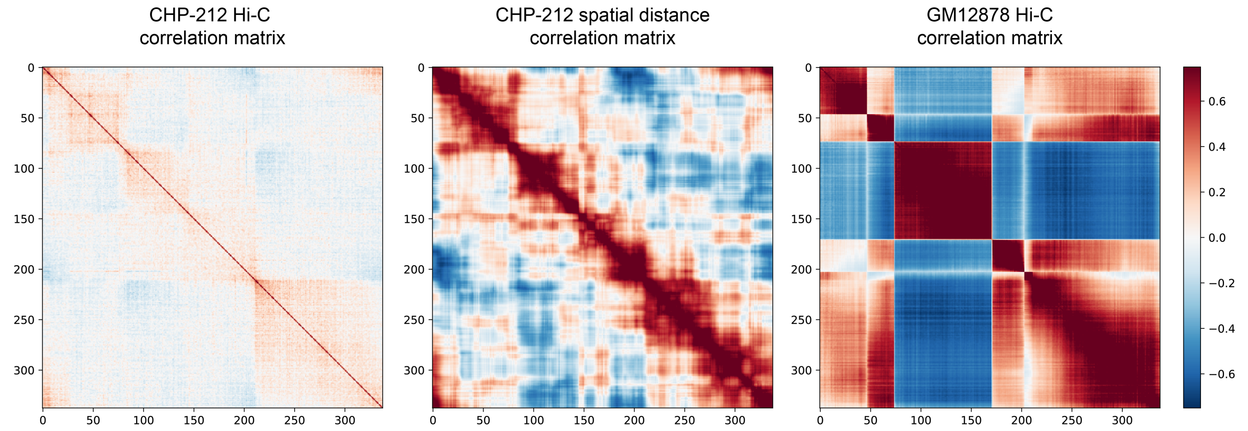

Supplementary Figure 17: Correlation matrices for generating A/B compartments on CHP-212 ecDNA. The first two matrices were obtained from Hi-C and from ec3D reconstructed spatial distances, respectively. The last matrix was obtained from GM12878 Hi-C as a control, where (5Kb) genomic bins were rearranged to match the ecDNA sequence. Color scale represents correlation coefficients ranging from -1 (blue) to +1 (red), with white indicating no correlation (0). Source data are provided as a Source Data file.

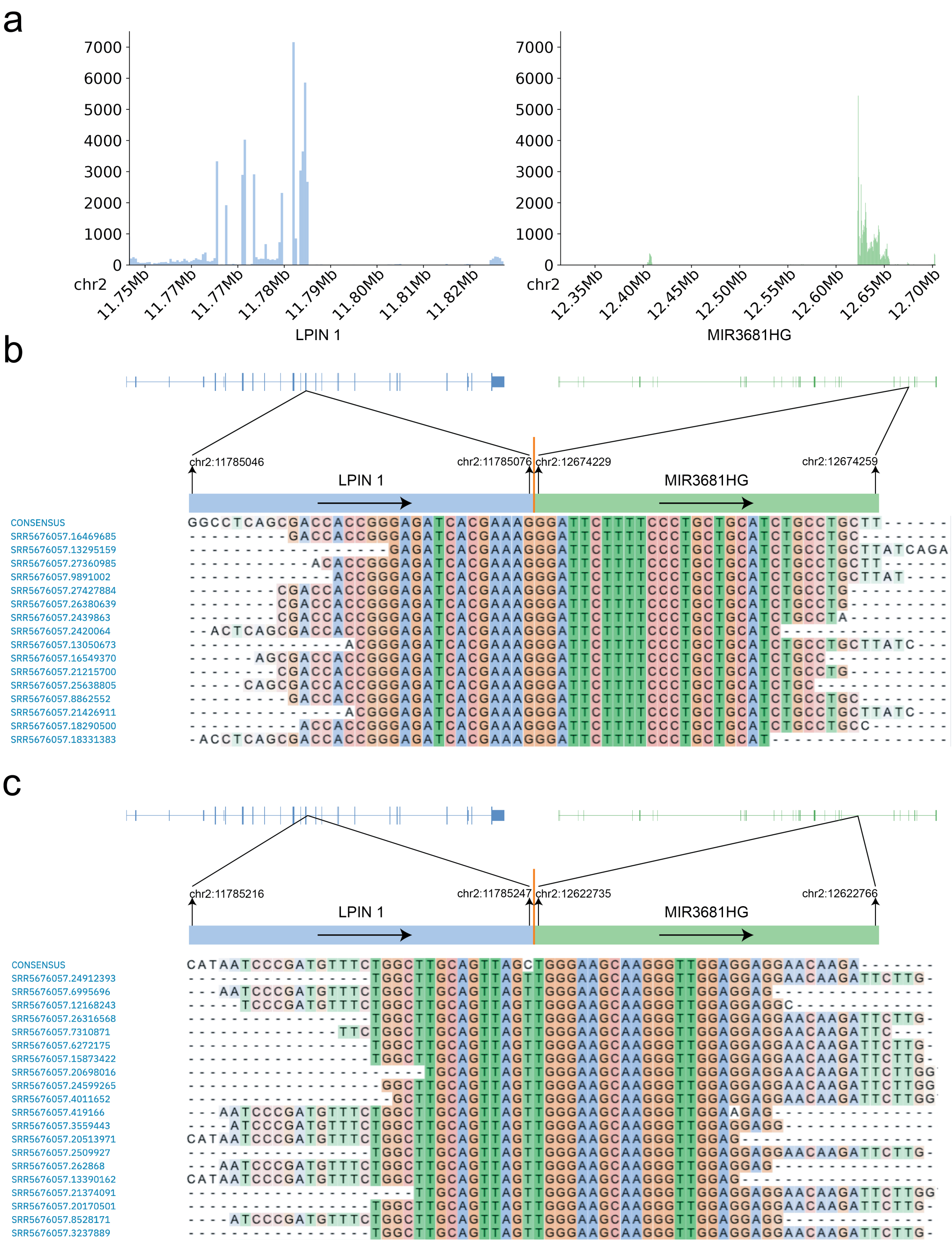

Supplementary Figure 18: Fusion transcript and readthrough event in the CHP-212 ecDNA. a, RNA-seq read coverage (y-axis) per 500 bp on *LPIN1* and *MIR3681HG* regions (x-axis). **b,** *LPIN1-MIR3681HG* fused transcript with supporting reads. **c,** Readthrough event with supporting reads. Source data are provided as a Source Data file.

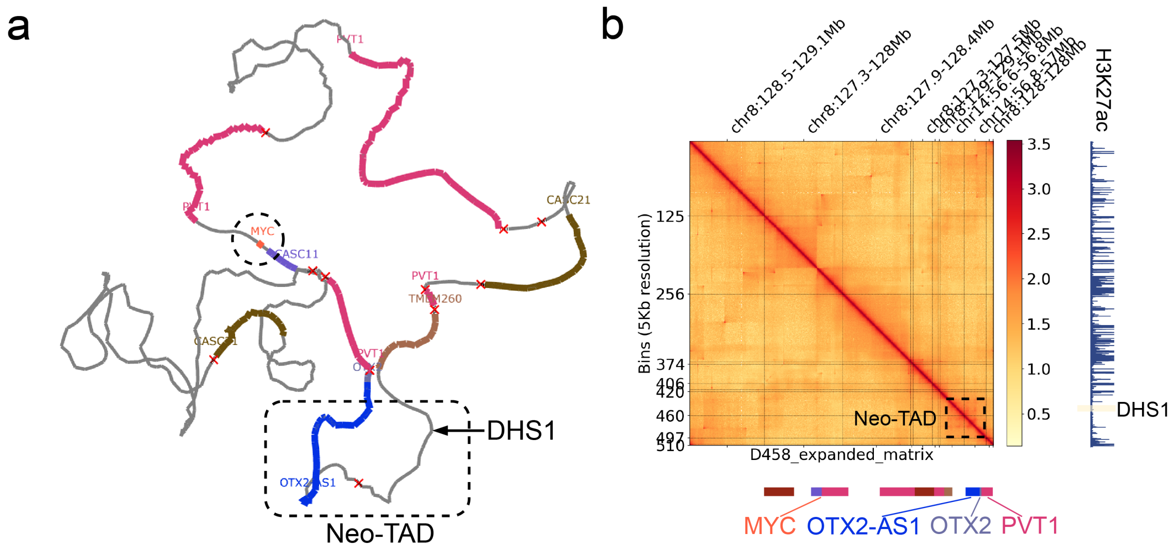

Supplementary Figure 19: Ec3D reconstruction of D458 (a) better clarifies the (sub)structure of a neo-TAD (b). Color scale in (b) represents $\log_{10}$-transformed normalized interaction frequencies. The neo-TAD involves an inversion of a segment 56.8-57Mb from chr14, which brings together a distal enhancer DHS1 and *OTX2*, but not *MYC* as suggested by ec3D.

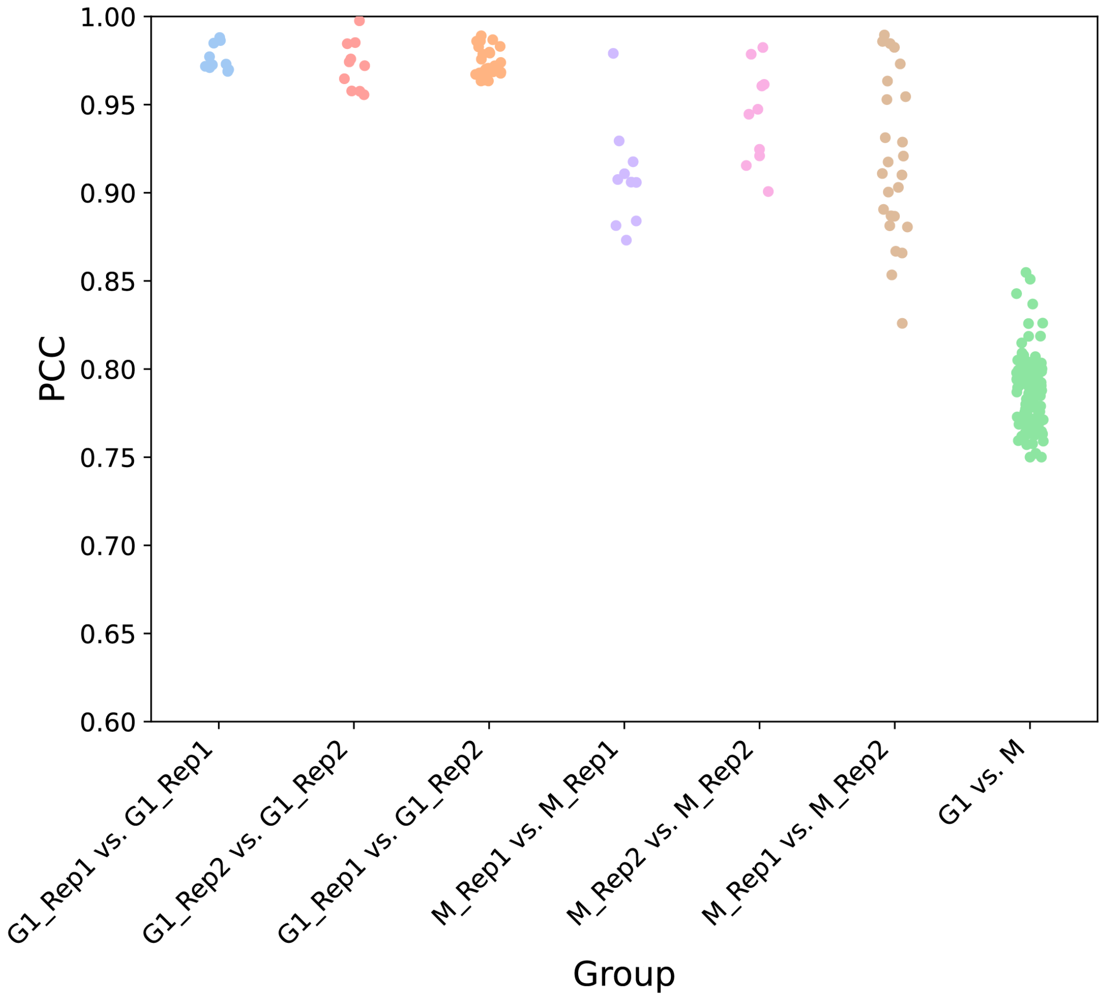

Supplementary Figure 20: Pearson's correlation coefficents (PCC) of distances between pairs of ensemble structures of the MSTO211H ecDNA. There are 2 Hi-C replicates for G1 phase and 2 replicates for M phase. An ensemble of 5 structures was generated for each replicate. Groups with the same replicate and phase contain 10 pairs of structures; groups with different replicates but the same phase contain 25 pairs of structures; the last group with different phases contains 100 pairs of structures. Source data are provided as a Source Data file.

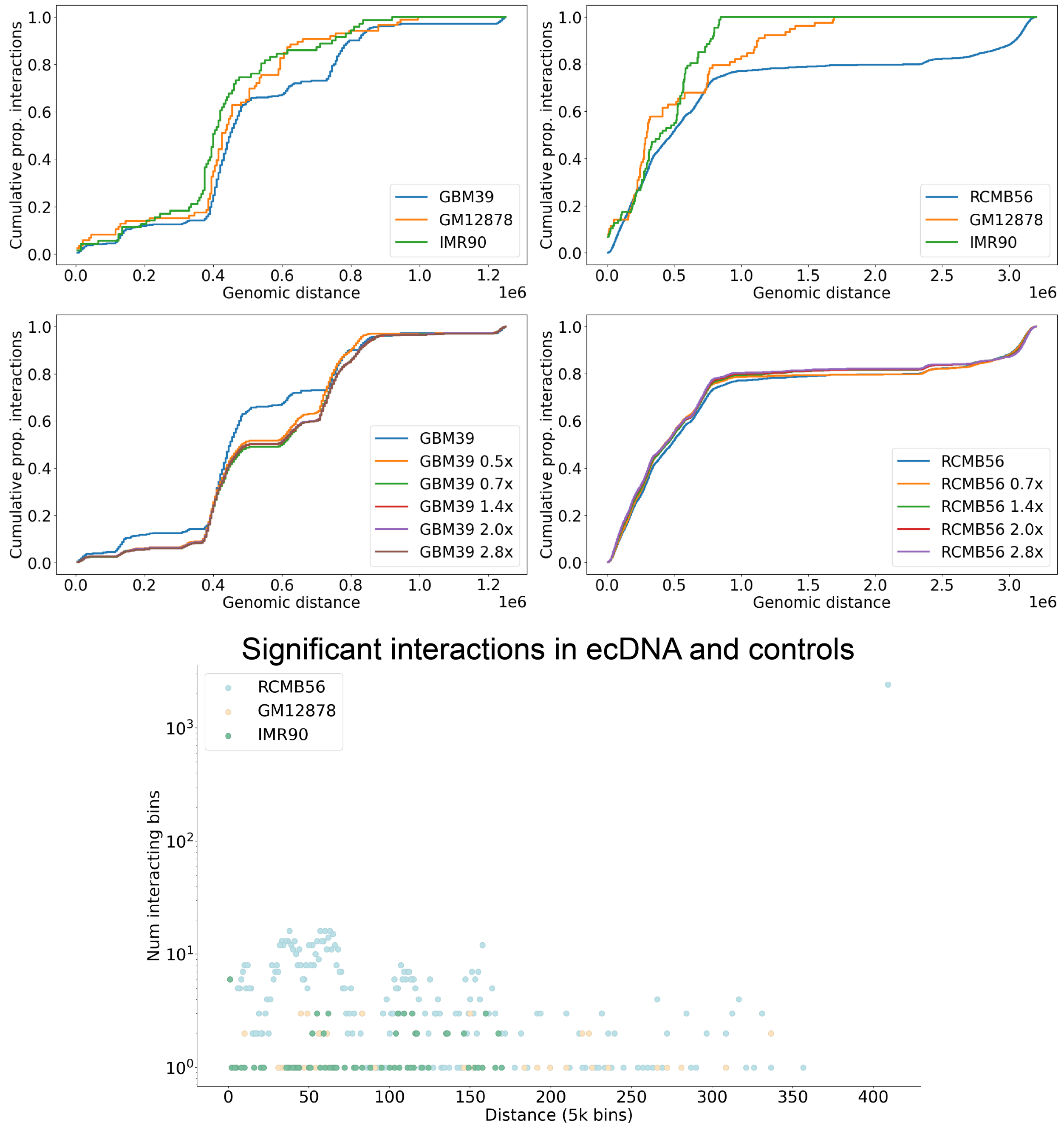

Supplementary Figure 21: Distributions of significant interactions with respect to genomic distances. The top four panels show cumulative distributions of significant interactions (ref-SI, y-axis) as a function of increasing genomic distance (x-axis). Two panels at the top show that both GBM39 and RCMB56 had more long-range interactions than controls GM12878 and IMR90, corresponding to slower increases in the cumulative distributions. Two panels in the middle show similar trends of significant interactions with upscaled and downscaled matrices. Note that in RCMB56, downscaling to 0.5x breaks the negative binomial property in most of the genomic distances, and as such, we omitted that curve. The bottom panel shows the distribution of number of significant interactions (ref-SI) as a function of reference genomic distances in RCMB56 ecDNA, compared to control cell lines GM12878 and IMR90.

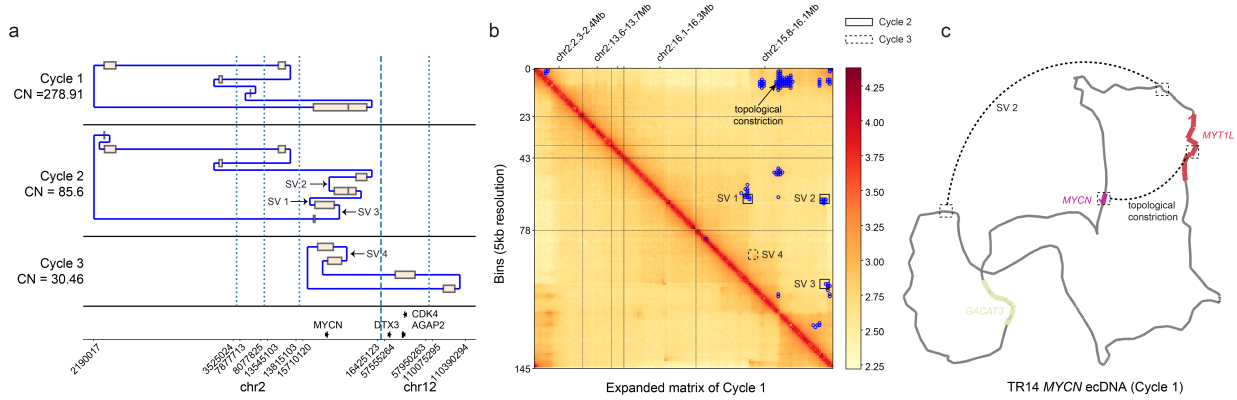

Supplementary Figure 22: Heterogeneity of ecDNA sequence in TR14. a, Sequences of three ecDNA cycles that amplify *MYCN* with different copy numbers in cell line TR14. **b,** Ec3D derived Hi-C matrix for Cycle 1. Color scale represents $\log_{10}$-transformed normalized interaction frequencies. Blue dots represent significant interactions (circ-SI) identified by ec3D. The arrow points to significant interactions due to topological constriction in Cycle 1, and black boxes (solid and dashed) indicate positions of unique SVs from Cycles 2 and 3. **c,** 3D structure of Cycle 1 with the topological constriction and SV 2 from (b). Source data are provided as a Source Data file.

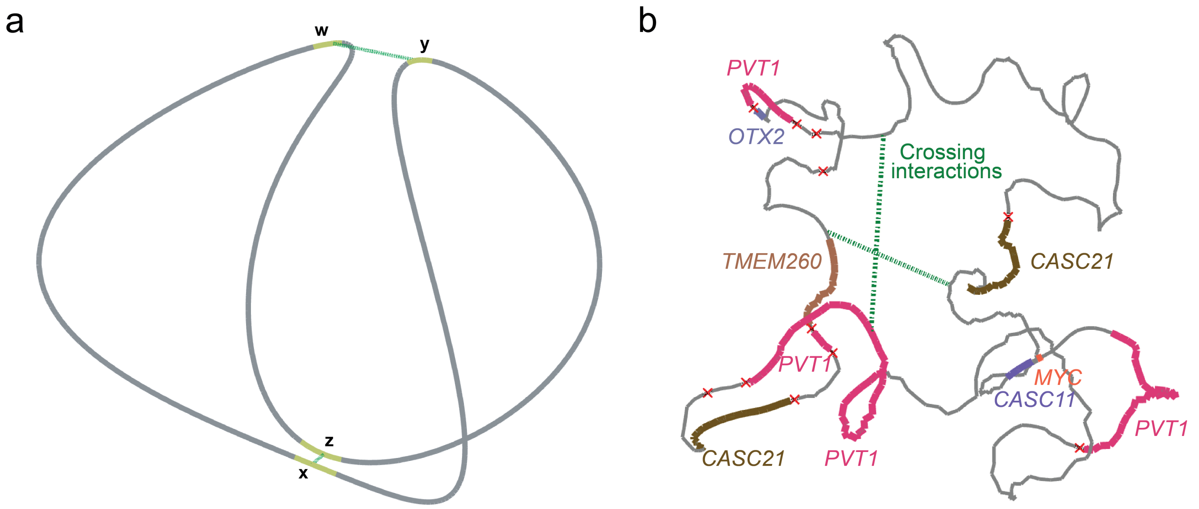

Supplementary Figure 23: "Crossing" interactions in 3D structures. a, Cartoon illustration of "crossing" interactions in a 3D structure comprising two topological constrictions (TCs). **b,** Representative crossing interactions on the 3D structure of D458 ecDNA.

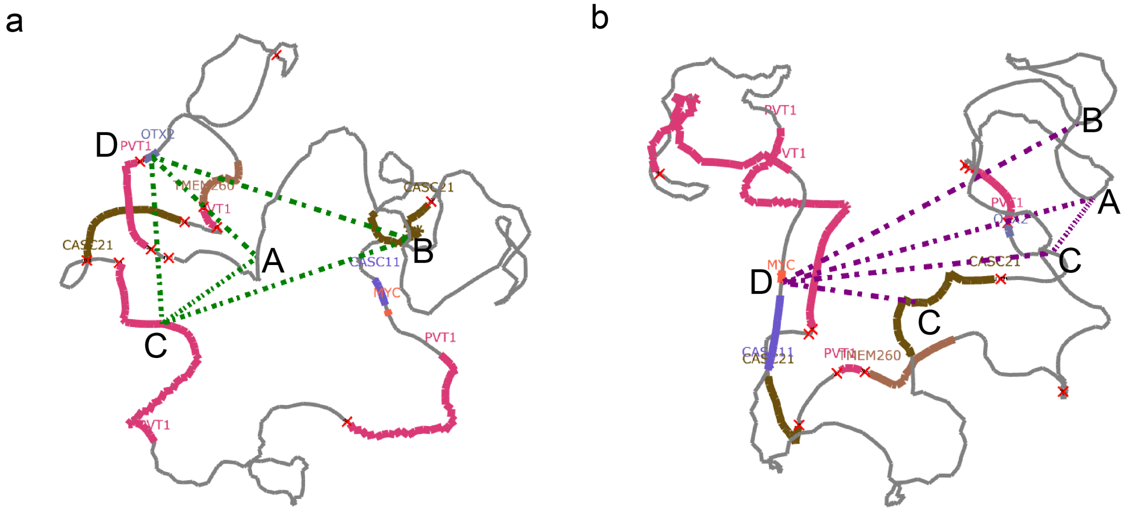

Supplementary Figure 24: The clique-like interactions (a) involving chr8 and chr14; and the star-like interactions (b) centered at MYC/PVT1 locus, in D458 ecDNA.

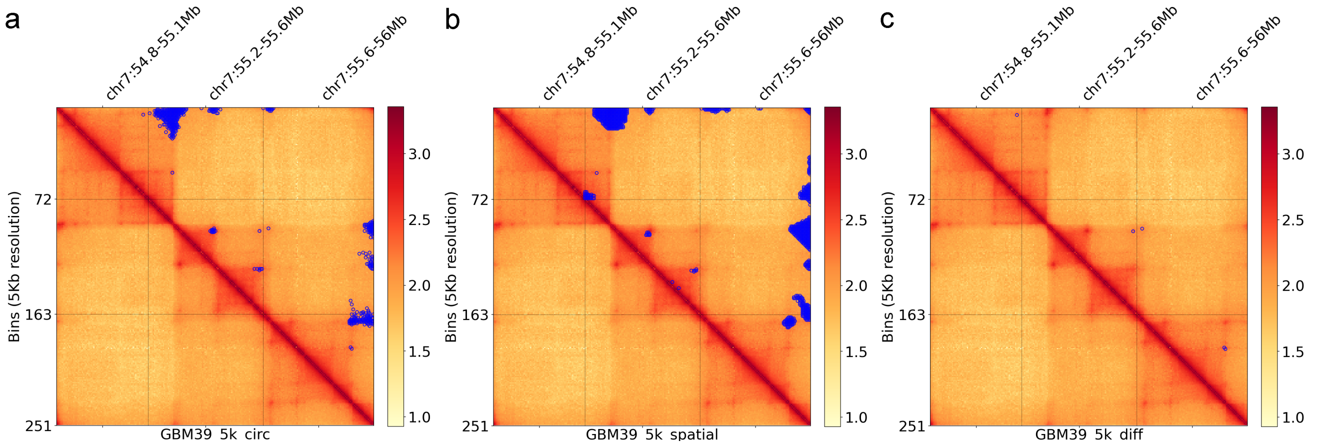

Supplementary Figure 25: Differential interactions in GBM39. Blue dots represent significant interactions identified by ec3D, with varying methods. Color scale represents $\log_{10}$-transformed normalized interaction frequencies. There are no obvious differences (c) between circ-SI (a) and spatial-SI (b), except a few loops (i.e., TAD boundaries). Source data are provided as a Source Data file.

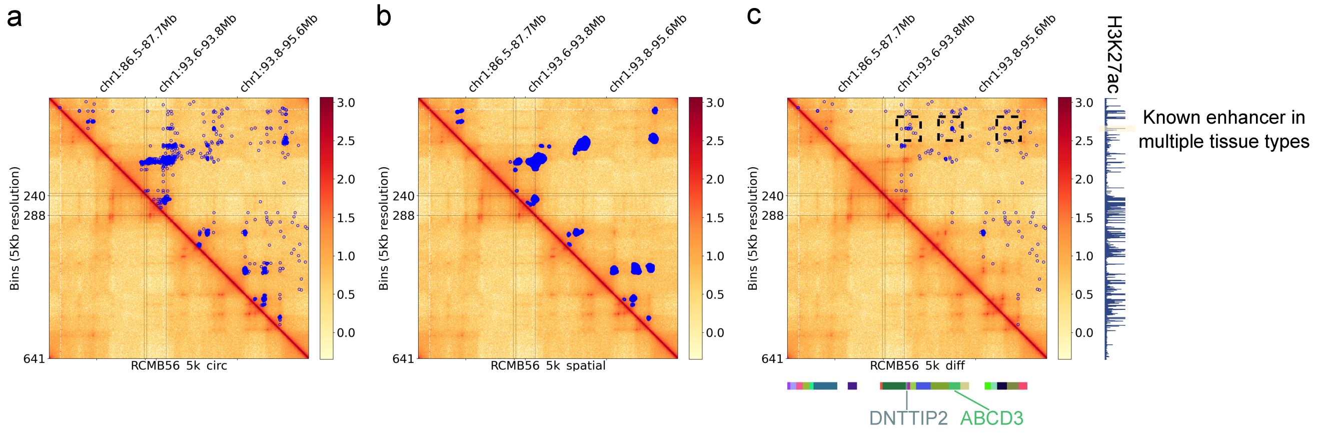

Supplementary Figure 26: Differential interactions in RCMB56. Blue dots represent significant interactions identified by ec3D, with varying methods. Color scale represents $\log_{10}$-transformed normalized interaction frequencies. The most remarkable differential interactions (c) that occur in circ-SI (a) but not spatial-SI (b) all involve an active enhancer at SELENOF locus. These interactions suggest potential *trans* interactions, due to the lack of spatial proximity in our 3D structure reconstruction. Source data are provided as a Source Data file.

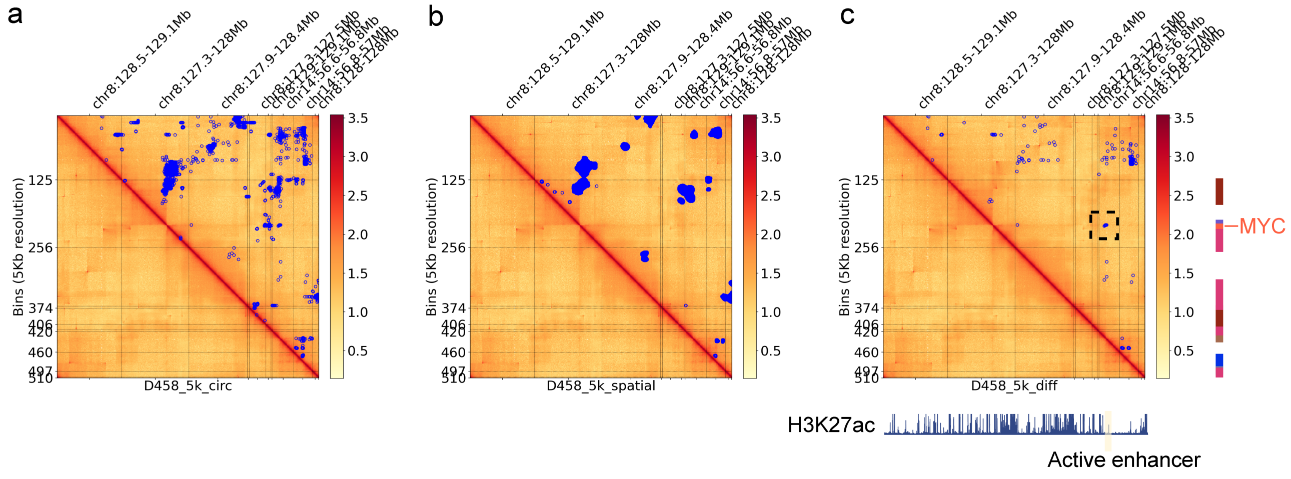

Supplementary Figure 27: Differential interactions in D458, excluding interactions from all duplicated segments. Blue dots represent significant interactions identified by ec3D, with varying methods. Color scale represents $\log_{10}$-transformed normalized interaction frequencies. The most remarkable differential interactions (c) that occur in circ-SI (a) but not spatial-SI (b) are between the MYC locus on chr8 and some enhancer region on chr14. These interactions suggest potential *trans* interactions, due to the lack of spatial proximity in our 3D structure reconstruction. Source data are provided as a Source Data file.

Supplementary Methods for Reconstructing the three-dimensional architecture of extrachromosomal DNA with ec3D

Simulations

Base structures. We simulated three circular base structures with k ($k \in \{1, 2, 3\}$) topological constrictions. Each base structure is defined by a set of parametric functions with a variable $\theta \in [0, 2\pi]$ and hyperparameter $p \in [0.90, 0.99]$ that controls the spatial distance (and interaction frequency) of the constrictions. If p is high, the constrictions have small spatial distances and strong interactions; If p is low, the constrictions have large spatial distances and weak interactions.

- $k = 1$
  - $x = f(\theta) = \cos\theta$
  - $y = g(\theta) = \sin\theta - p\sin^4\theta$ if $\sin\theta > 0$ else $\sin\theta + p\sin^4\theta$
  - $z = h(\theta) = \cos^2\theta$
- $k = 2$
  - $x = f(\theta) = \cos\theta - p\cos^4\theta$ if $\cos\theta > 0$ else $\cos\theta + p\cos^4\theta$
  - $y = g(\theta) = \sin\theta - p\sin^4\theta$ if $\sin\theta > 0$ else $\sin\theta + p\sin^4\theta$
  - $z = h(\theta) = \sin^2\theta$
- $k = 3$
  - $x = f(\theta) = \cos\theta \cdot \frac{1}{4}(1 + p\cos 3\theta)$
  - $y = g(\theta) = \sin\theta \cdot \frac{1}{4}(1 + p\cos 3\theta)$
  - $z = h(\theta) = \frac{1}{4}\sin 3\theta$

Given a set of parametric functions f, g, h, we generated 10,000 3D points with $\theta_i = \frac{i}{10000} \cdot 2\pi$, where $i = 0, 1, \cdots, 9999$. We then calculated the arc length between point 0 and $i > 0$ by $l_i = \int_0^{\theta_i} \sqrt{f'^2(\theta) + g'^2(\theta) + h'^2(\theta)}d\theta$, which gave us 10,000 data points (l_i, θ_i). We used SciPy to interpolate the 10,000 data points to find a mapping F from the arc length l_i to the parameter θ_i, i.e., $\theta_i = F(l_i)$. As a result, we can generate N points that are evenly spaced on the 3D structure by calculating $\theta_i = F(\frac{i}{n}L)$ for $i = 0, 1, \cdots, N$, where L is the total arc length, i.e., $L = \int_0^{2\pi} \sqrt{f'^2(\theta) + g'^2(\theta)2 + h'^2(\theta)}d\theta$.

Local folds. Given adjacent points X_i and X_{i+1} on a base structure, we added local folds between the two points by random walks. We denote the difference between the two points as a vector $\mathbf{d} = X_{i+1} - X_i$. If we want to generate a local fold with m points, where m is an even number, then we have the average distance vector $\Delta = \frac{2}{m} \cdot \mathbf{d}$. Given the step length $l = \frac{L}{N}$, we can generate a step vector $\mathbf{s} = (l\sin\theta\cos\phi, l\sin\theta\sin\phi, l\cos\phi)$ by randomly sampling θ and ϕ from the range $[0, 2\pi]$. To guarantee that the local fold starts from X_i and ends at X_{i+1}, we need to generate

a complementary step vector $\mathbf{s}' = \Delta - \mathbf{s}$. As a result, we can generate a set of m step vectors $S = \{\mathbf{s}_1, \mathbf{s}_2, \cdots, \mathbf{s}_m\}$, where $\mathbf{s}_j + \mathbf{s}_{m/2+j} = \Delta, \forall j \in \{1, 2, \cdots, m/2\}$. To generate a local fold with m points, we can simply add the accumulated sum of step vectors to the starting point X_i, i.e., $P = \{X_i + \sum_{k=1}^{j} \mathbf{s}_k | j = 1, 2, \cdots, m\}$. Note that $X_i + \sum_{\mathbf{s} \in S} \mathbf{s} = X_{i+1}$ since $\mathbf{d} = \sum_{\mathbf{s} \in S} \mathbf{s}$. In this way, we generated various local folds with m points, where $m \in \{16, 18, 20, 22\}$, and the adjacent local folds 4 or $22 + 4(k-1)$ points in between (k is the number of topological constrictions). The smaller value makes adjacent local folds form as a TAD; the greater value makes two local folds far apart.

Hi-C simulation. Given a 3D structure, we considered the evenly spaced points as equally sized bins in the Hi-C data. We simulated pairwise Hi-C interaction frequencies by randomly sampling integers from the Poisson distribution with mean $\beta \cdot d_{ij}^{\alpha}$, where d_{ij} represents the Euclidean distance between bin i and bin j. Furthermore, we simulated duplication by designating n ($n \in \{1, 2, \cdots, \texttt{ceil}(N_e/10)\}$) pairs of local folds as duplicated regions. We then summed up the Hi-C interactions of all duplicated bins, assigned the results to the copy with the least index, and removed the other copies in the Hi-C matrix. As a result, we simulated 450 collapsed Hi-C and 450 expanded Hi-C matrices.
