# Supplementary material for: Reconstructing the three-dimensional architecture of extrachromosomal DNA with ec3D
Source: Nat Commun. 2025 Dec 20;17:894. doi: 10.1038/s41467-025-67614-7 (PMC12830899; doi:10.1038/s41467-025-67614-7)
Supplement: Supplementary file 2 — Description of Additional Supplementary Information [file 41467_2025_67614_MOESM2_ESM.pdf]

Description of Additional Supplementary Files

File Name: Supplementary Data 1
Description: RMSD and PCC ranges from simulated Hi-C. RMSD and PCC between the 900 (450 without duplication and 450 with duplication) ground truth and reconstructed structures were compared to those computed with two randomly selected structures, with the same or different base structure(s).

File Name: Supplementary Data 2
Description: Statistical difference, through a two-sided Wilcoxon rank-sum test, between the RMSD and PCC values from ec3D's reconstruction and two random structures.

File Name: Supplementary Data 3
Description: Summary characteristics of the 9 cell lines used in this study.

File Name: Supplementary Data 4
Description: Genomic segments, in order, composing the ecDNA/HSR cycles in 9 cancer cell lines.

File Name: Supplementary Data 5
Description: RMSD and PCC values computed from local structures of duplicated segments reconstructed by ec3D, and their empirical P-values from a permutation test.

File Name: Supplementary Data 6
Description: Summary of methods to compute significant interactions, and the types of significant interactions they can capture. Dark grey indicates that the corresponding method for sure captures that cause of significant interactions; Light grey indicates that we do not intend to capture such interactions, but they can still play a role in our computation of significant interactions.

File Name: Supplementary Data 7
Description: TAD boundaries (from 3D structure) matched to CTCF peaks on CHP-212 ecDNA. TAD boundaries and CTCF peaks are represented by 5-kb bins on the ecDNA.

File Name: Supplementary Data 8
Description: TAD boundaries (from HiC) matched to CTCF peaks on CHP-212 ecDNA. TAD boundaries and CTCF peaks are represented by 5-kb bins on the ecDNA.

File Name: Supplementary Data 9
Description: Clusters of significant interactions (ref-SI) in D458. Clusters with single interactions are omitted.

File Name: Supplementary Data 10
Description: *MDM2* Oligopaints

File Name: Supplementary Data 11
Description: *MDM2* IDT Oligos
