# Supplementary material for: Reconstructing the three-dimensional architecture of extrachromosomal DNA with ec3D
Source: Nat Commun. 2025 Dec 20;17:894. doi: 10.1038/s41467-025-67614-7 (PMC12830899; doi:10.1038/s41467-025-67614-7)
Supplement: Supplementary file 14 — Reporting Summary [file 41467_2025_67614_MOESM14_ESM.pdf]

nature portfolio

Corresponding author(s): Vineet Bafna

Last updated by author(s): Oct 24, 2025

Reporting Summary

Nature Portfolio wishes to improve the reproducibility of the work that we publish. This form provides structure for consistency and transparency in reporting. For further information on Nature Portfolio policies, see our Editorial Policies and the Editorial Policy Checklist.

Statistics

For all statistical analyses, confirm that the following items are present in the figure legend, table legend, main text, or Methods section.

n/a	Confirmed	
☐	☒	The exact sample size (*n*) for each experimental group/condition, given as a discrete number and unit of measurement
☒	☐	A statement on whether measurements were taken from distinct samples or whether the same sample was measured repeatedly
☐	☒	The statistical test(s) used AND whether they are one- or two-sided<br>*Only common tests should be described solely by name; describe more complex techniques in the Methods section.*
☒	☐	A description of all covariates tested
☐	☒	A description of any assumptions or corrections, such as tests of normality and adjustment for multiple comparisons
☒	☐	A full description of the statistical parameters including central tendency (e.g. means) or other basic estimates (e.g. regression coefficient) AND variation (e.g. standard deviation) or associated estimates of uncertainty (e.g. confidence intervals)
☐	☒	For null hypothesis testing, the test statistic (e.g. *F*, *t*, *r*) with confidence intervals, effect sizes, degrees of freedom and *P* value noted<br>*Give P values as exact values whenever suitable.*
☒	☐	For Bayesian analysis, information on the choice of priors and Markov chain Monte Carlo settings
☒	☐	For hierarchical and complex designs, identification of the appropriate level for tests and full reporting of outcomes
☐	☒	Estimates of effect sizes (e.g. Cohen's *d*, Pearson's *r*), indicating how they were calculated

Our web collection on statistics for biologists contains articles on many of the points above.

Software and code

Policy information about availability of computer code

Data collection	We downloaded whole genome sequencing (WGS) data for GBM39, GBM39HSR, RCMB56, IMR-5/75, CHP-212, TR14 and MSTO-211H cell lines; OGM contigs for D458; Hi-C data for RCMB56, D458, IMR-5/75, CHP-212, TR14 and MSTO-211H; RNA-seq, single cell RNA-seq and CTCF ChIP-seq data for CHP-212 from previously published studies, as described below in the "availability of data" section. We downloaded processed high coverage GM12878 and IMR90 Hi-C, in *.mcool format, from 4D nucleome (https://data.4dnucleome.org/).<br><br>H2170: EcDNA genome reconstruction was performed in this study. Data are provided in Supplementary Data 4.<br><br>The Hi-C library for H2170 was prepared using the Arima-HiC kit. Hi-C libraries for GBM39EC and GBM39HSR were prepared following a standard protocol to investigate chromatin interactions. Samples were sequenced using Illumina NovaSeq in 150 bp paired-end reads, with 3 replications for both GBM39EC and GBM39HSR. We combined all Hi-C replications (for each sample) into a single matrix in our structural reconstruction.
Data analysis	ec3D (https://github.com/AmpliconSuite/ec3d)<br>bwa version 0.7.17-r1188 (https://github.com/lh3/bwa)<br>minimap2 version 2.24-r1122 (https://github.com/lh3/minimap2)<br>HiC-Pro version 3.1.0 (https://github.com/nservant/HiC-Pro)<br>cooler version 0.8.2 (https://github.com/open2c/cooler)<br>iced (https://github.com/hiclib/iced)<br>hic2cool version 1.0.1 (https://github.com/4dn-dcic/hic2cool)<br>CoRAL (https://github.com/AmpliconSuite/CoRAL)

AmpliconArchitect version 1.3.r5 (https://github.com/AmpliconSuite/AmpliconArchitect)
AmpliconReconstructor version 1.01 (https://github.com/AmpliconSuite/AmpliconReconstructorOM)
STAR-Fusion version 1.14.0 (https://github.com/STAR-Fusion/STAR-Fusion)
deepTools version 3.5.6 (https://deeptools.readthedocs.io/en/latest/)
Clustal Omega version 1.2.4 (http://www.clustal.org/omega/)
FAN-C version 0.9.28 (https://github.com/vaquerizaslab/fanc)
MiniMDS (https://github.com/seqcode/miniMDS)
ShRec3D (https://github.com/kpj/ShRec3D)
PASTIS version 0.5.0 (https://github.com/hiclib/pastis)
OligoLego (https://github.com/gnir/OligoLego)

For manuscripts utilizing custom algorithms or software that are central to the research but not yet described in published literature, software must be made available to editors and reviewers. We strongly encourage code deposition in a community repository (e.g. GitHub). See the Nature Portfolio guidelines for submitting code & software for further information.

Data

Policy information about availability of data

All manuscripts must include a data availability statement. This statement should provide the following information, where applicable:

- Accession codes, unique identifiers, or web links for publicly available datasets
- A description of any restrictions on data availability
- For clinical datasets or third party data, please ensure that the statement adheres to our policy

Long read WGS data for GBM39 and GBM39HSR are available under SRA accession PRJNA1110283. Long read WGS, Hi-C and CTCF ChIP-seq data for CHP-212 and IMR-5/75 are available under SRA accession PRJNA622577; RNA-seq data of CHP-212 are available under GEO accession GSE90683. Short read WGS data for RCMB56 (PDX) and OGM contigs for D458 are available under SRA accession PRJNA1011359. Hi-C data for RCMB56 and D458 are available under GEO accession GSE240985. Hi-C data for H2170, GBM39 and GBM39HSR are available under SRA accession PRJNA1259562. Long read WGS for TR14 are available under SRA accession PRJNA670737. Hi-C data for TR14 are available under SRA accession PRJNA732417. Short read WGS and Hi-C data for MSTO-211H are available under SRA accession PRJNA1263546. The G&T single-cell data for CHP212 are available on the European Genome Archive (EGA) under accession number EGAS50000000509.

Research involving human participants, their data, or biological material

Policy information about studies with human participants or human data. See also policy information about sex, gender (identity/presentation), and sexual orientation and race, ethnicity and racism.

Reporting on sex and gender	N/A
Reporting on race, ethnicity, or other socially relevant groupings	N/A
Population characteristics	N/A
Recruitment	N/A
Ethics oversight	N/A

Note that full information on the approval of the study protocol must also be provided in the manuscript.

Field-specific reporting

Please select the one below that is the best fit for your research. If you are not sure, read the appropriate sections before making your selection.

[x] Life sciences [ ] Behavioural & social sciences [ ] Ecological, evolutionary & environmental sciences

For a reference copy of the document with all sections, see nature.com/documents/nr-reporting-summary-flat.pdf

Life sciences study design

All studies must disclose on these points even when the disclosure is negative.

Sample size	We reconstructed 900 simulated ecDNA structures and ecDNAs from 9 cancer cell lines or PDXs. For simulation, we generated 10 structures for each combination of k = 1, 2, 3 topological constrictions (i.e., major conformations) and N_e = 250, 500, 750 bins, with random local folds and spatial distances between the two intervals participating in each topological constriction, resulting in 90 structures in total. For each simulated structure, we generated 5 matrices without duplication and 5 matrices with duplication, with random α and β parameters, giving the 900 simulated Hi-C matrices in total.
Data exclusions	No data were excluded from the analyses.
Replication	This is a computational study based on (re)analysis of existing WGS and Hi-C datasets; and simulated data. All analyses were performed using documented code available in our GitHub repository. The algorithm was tested on multiple independent datasets (RCMB56, D458, GBM39, GBM39HSR, IMR-5/75, CHP-212, H2170, MSTO-211H, and TR14 cell lines) with consistent performance across all samples, as well as a simulation framework that generates reproducible synthetic datasets using selected parameters. The simulation code and parameter sets are

	also provided to enable independent verification.
Randomization	The experiments were not randomized as they involved computational analysis of existing datasets.
Blinding	N/A

Reporting for specific materials, systems and methods

We require information from authors about some types of materials, experimental systems and methods used in many studies. Here, indicate whether each material, system or method listed is relevant to your study. If you are not sure if a list item applies to your research, read the appropriate section before selecting a response.

Materials & experimental systems

n/a	Involved in the study	
☒	☐	Antibodies
☐	☒	Eukaryotic cell lines
☒	☐	Palaeontology and archaeology
☐	☒	Animals and other organisms
☒	☐	Clinical data
☒	☐	Dual use research of concern
☒	☐	Plants

Methods

n/a	Involved in the study	
☐	☒	ChIP-seq
☒	☐	Flow cytometry
☒	☐	MRI-based neuroimaging

Eukaryotic cell lines

Policy information about cell lines and Sex and Gender in Research

Cell line source(s)	We reused the data from the following studies. Descriptions of cell lines can be found in the relevant reporting summaries:<br>GBM39, GBM39HSR: Turner et al. 2017 (https://doi.org/10.1038/nature21356) and Wu et al. 2019 (https://doi.org/10.1038/s41586-019-1763-5) ;<br>IMR-5/75, CHP-212: Helmsauer et al. 2020 (https://doi.org/10.1038/s41467-020-19452-y);<br>D458: Chapman et al. 2023 (https://doi.org/10.1038/s41588-023-01551-3);<br>TR14: Hung et al. 2021 (https://doi.org/10.1038/s41586-021-04116-8);<br>MSTO-211H: Xie et al. 2025 (https://doi.org/10.1101/2024.12.31.630921).<br><br>H2170 was purchased from ATCC.
Authentication	Identity of TR14, IMR-5/75 and CHP-212 were verified by STR genotyping;<br>Other cell lines were not authenticated.
Mycoplasma contamination	All cell-lines were tested negative for mycoplasma.
Commonly misidentified lines<br>(See ICLAC register)	No commonly misidentified lines were used in this study.

Animals and other research organisms

Policy information about studies involving animals; ARRIVE guidelines recommended for reporting animal research, and Sex and Gender in Research

Laboratory animals	We reused the data generated by Chapman et al. 2023 (https://doi.org/10.1038/s41588-023-01551-3)
Wild animals	*Provide details on animals observed in or captured in the field; report species and age where possible. Describe how animals were caught and transported and what happened to captive animals after the study (if killed, explain why and describe method; if released, say where and when) OR state that the study did not involve wild animals.*
Reporting on sex	*Indicate if findings apply to only one sex; describe whether sex was considered in study design, methods used for assigning sex. Provide data disaggregated for sex where this information has been collected in the source data as appropriate; provide overall numbers in this Reporting Summary. Please state if this information has not been collected. Report sex-based analyses where performed, justify reasons for lack of sex-based analysis.*
Field-collected samples	*For laboratory work with field-collected samples, describe all relevant parameters such as housing, maintenance, temperature, photoperiod and end-of-experiment protocol OR state that the study did not involve samples collected from the field.*
Ethics oversight	*Identify the organization(s) that approved or provided guidance on the study protocol, OR state that no ethical approval or guidance was required and explain why not.*

Note that full information on the approval of the study protocol must also be provided in the manuscript.

Plants

Seed stocks	*Report on the source of all seed stocks or other plant material used. If applicable, state the seed stock centre and catalogue number. If plant specimens were collected from the field, describe the collection location, date and sampling procedures.*
Novel plant genotypes	*Describe the methods by which all novel plant genotypes were produced. This includes those generated by transgenic approaches, gene editing, chemical/radiation-based mutagenesis and hybridization. For transgenic lines, describe the transformation method, the number of independent lines analyzed and the generation upon which experiments were performed. For gene-edited lines, describe the editor used, the endogenous sequence targeted for editing, the targeting guide RNA sequence (if applicable) and how the editor was applied.*
Authentication	*Describe any authentication procedures for each seed stock used or novel genotype generated. Describe any experiments used to assess the effect of a mutation and, where applicable, how potential secondary effects (e.g. second site T-DNA insertions, mosiacism, off-target gene editing) were examined.*

ChIP-seq

Data deposition

☒ Confirm that both raw and final processed data have been deposited in a public database such as GEO.

☒ Confirm that you have deposited or provided access to graph files (e.g. BED files) for the called peaks.

Data access links<br>*May remain private before publication.*	We reused the data generated by Boeva et al. 2017 (https://doi.org/10.1038/ng.3921)
Files in database submission	*Provide a list of all files available in the database submission.*
Genome browser session<br>(e.g. UCSC)	*Provide a link to an anonymized genome browser session for "Initial submission" and "Revised version" documents only, to enable peer review. Write "no longer applicable" for "Final submission" documents.*

Methodology

Replicates	See Boeva et al. 2017 (https://doi.org/10.1038/ng.3921)
Sequencing depth	*Describe the sequencing depth for each experiment, providing the total number of reads, uniquely mapped reads, length of reads and whether they were paired- or single-end.*
Antibodies	*Describe the antibodies used for the ChIP-seq experiments; as applicable, provide supplier name, catalog number, clone name, and lot number.*
Peak calling parameters	*Specify the command line program and parameters used for read mapping and peak calling, including the ChIP, control and index files used.*
Data quality	*Describe the methods used to ensure data quality in full detail, including how many peaks are at FDR 5% and above 5-fold enrichment.*
Software	*Describe the software used to collect and analyze the ChIP-seq data. For custom code that has been deposited into a community repository, provide accession details.*
